# *Col6a1* Null Mice as a Model to Study Skin Phenotypes in Patients with Collagen VI Related Myopathies: Expression of Classical and Novel Collagen VI Variants during Wound Healing

**DOI:** 10.1371/journal.pone.0105686

**Published:** 2014-08-26

**Authors:** Sandra Lettmann, Wilhelm Bloch, Tobias Maaß, Anja Niehoff, Jan-Niklas Schulz, Beate Eckes, Sabine A. Eming, Paolo Bonaldo, Mats Paulsson, Raimund Wagener

**Affiliations:** 1 Center for Biochemistry, Medical Faculty, University of Cologne, Cologne, Germany; 2 Institute of Cardiovascular Research and Sport Medicine, German Sport University, Cologne, Germany; 3 Institute of Biomechanics and Orthopaedics, German Sport University, Cologne, Germany; 4 Cologne Center for Musculoskeletal Biomechanics, Medical Faculty, University of Cologne, Cologne, Germany; 5 Department of Dermatology, University of Cologne, Cologne, Germany; 6 Center for Molecular Medicine Cologne, University of Cologne, Cologne, Germany; 7 Cologne Excellence Cluster on Cellular Stress Responses in Aging-Associated Diseases, University of Cologne, Cologne, Germany; 8 Department of Molecular Medicine, University of Padova, Padova, Italy; UMR CNRS 5242 - ENS de Lyon- Université Lyon 1, France

## Abstract

Patients suffering from collagen VI related myopathies caused by mutations in *COL6A1, COL6A2* and *COL6A3* often also display skin abnormalities, like formation of keloids or “cigarette paper” scars, dry skin, striae rubrae and keratosis pilaris (follicular keratosis). Here we evaluated if *Col6a1* null mice, an established animal model for the muscle changes in collagen VI related myopathies, are also suitable for the study of mechanisms leading to the skin pathology. We performed a comprehensive study of the expression of all six collagen VI chains in unwounded and challenged skin of wild type and *Col6a1* null mice. Expression of collagen VI chains is regulated in both skin wounds and bleomycin-induced fibrosis and the collagen VI α3 chain is proteolytically processed in both wild type and *Col6a1* null mice. Interestingly, we detected a decreased tensile strength of the skin and an altered collagen fibril and basement membrane architecture in *Col6a1* null mice, the latter being features that are also found in collagen VI myopathy patients. Although *Col6a1* null mice do not display an overt wound healing defect, these mice are a relevant animal model to study the skin pathology in collagen VI related disease.

## Introduction

Mutations in *COL6A1, COL6A2* and *COL6A3* encoding collagen VI, cause Ullrich congenital muscular dystrophy (UCMD), Bethlem myopathy (BM) and myosclerosis myopathy [1]–[3]. In addition to the obvious muscular phenotype many patients also display skin abnormalities, including a predisposition for keratosis pilaris (follicular keratosis), abnormal scarring with formation of keloids or “cigarette paper” scars, dry skin, and striae rubrae [4]–[8].

Collagen VI forms a distinct microfibrillar network in most forms of extracellular matrix that anchors interstitial structures, such as nerves, blood vessels and larger collagen fibrils. In addition to being a collagen it belongs to the superfamily of proteins containing von Willebrand factor A (VWA) domains [9], globular protein modules that act by mediating protein-protein interactions. Collagen VI was long considered to consist of three genetically distinct α-chains (α1, α2 and α3). These chains form heterotrimeric monomers that assemble into dimers and tetramers already in the cell [10], [11]. After secretion, polymers are formed by end-to-end interactions of the pre-assembled tetramers, yielding the characteristic beaded filaments seen by electron microscopy [12], [13].

More recently, three novel collagen VI α-chains, α4, α5, and α6, encoded by the distinct genes *Col6a4*, *Col6a5*, and *Col6a6* were identified [14], [15]. These chains are composed of seven N-terminal VWA domains, a collagen triple helical region and a C-terminal non-collagenous domain containing two or three C-terminal VWA domains and one or two unique sequences. In addition, the α4 chain carries a Kunitz domain. Their triple helical regions are most similar to that of the α3 chain, and, in general, the recently identified chains resemble this chain. In contrast to the α3 chain, the α4, α5 and α6 chains have highly restricted distributions often associated with basement membranes [16].

Collagen VI microfibril assembly is hampered in *Col6a1* null (*Col6a1^−/−^*) mice which display an early-onset muscle pathology that most closely resembles that of BM patients. These mice represent a valuable model for investigating the pathogenic mechanisms of collagen VI diseases at the molecular level and studies on *Col6a1* null mice revealed that mitochondrial dysfunction and defective autophagy are involved in the pathogenesis of collagen VI myopathies [17], [18]. We aimed to use the *Col6a1* null mouse strain as a model to study the role of collagen VI in the pathogenesis of skin abnormalities associated with collagen VI related myopathies. To evaluate if these mice adequately reflect the human skin phenotypes we performed a comprehensive study of the cutaneous expression of all six collagen VI chains, determined the skin morphology at the microscopic and ultrastructural levels, and studied wound healing. We compared our results to findings in wild type mice and related our observations to published data on the skin of collagen VI myopathy patients.

## Results

### Collagen VI deficiency does not impact skin morphology

Visual examination of the skin of collagen VI deficient mice revealed no obvious abnormalities and light microscope analysis showed a similar appearance of wild type and *Col6a1* null skin (Fig. S1a). This was surprising as not only the α1 chain is lacking in these mice, but the assembly and secretion of the other collagen VI chains is also severely affected (for details, see 3.3) Apparently collagen VI is not necessary to maintain the morphology of mouse skin under physiological conditions.

### Wound morphology in collagen VI deficient mice is not changed

Wound healing experiments were performed in skin of wild type and *Col6a1* null mice in which full thickness excisional defects had been created [19], [20]. Light microscope analysis of the wounds did not reveal any obvious differences between wild type and *Col6a1* null mice at day 4, 7, 10 and 14 after wounding (Fig. S1b). The distance between the severed ends of the panniculus carnosus and the area of the granulation tissue were unchanged (Fig. S2).

### Expression of collagen VI chains is regulated in skin wounds and fibrosis

**Figure 1:**
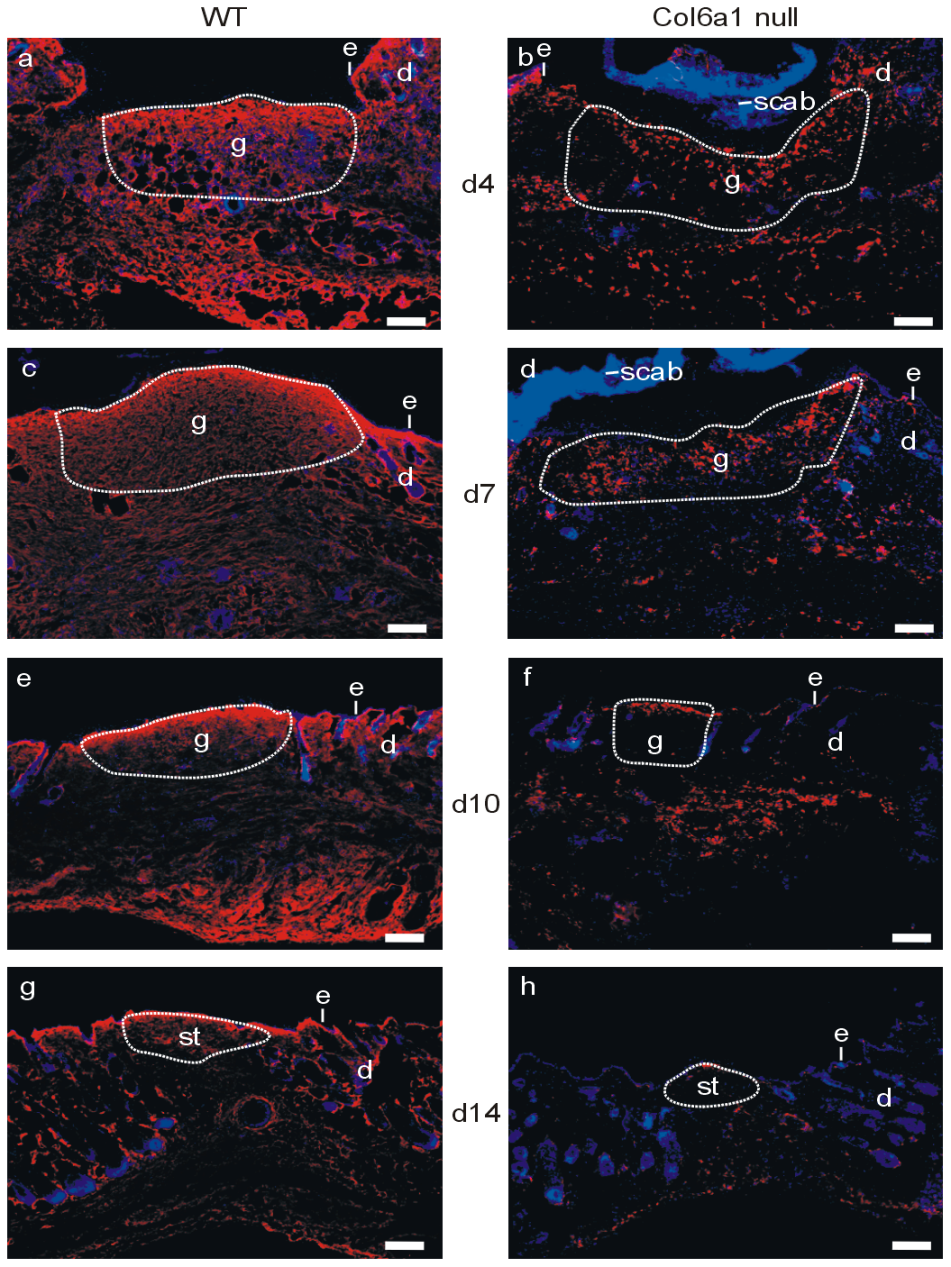
Collagen VI α3 expression during skin wound healing. Frozen sections of wounds of wild type and *Col6a1* null mice at 4, 7, 10 and 14 days after wounding were incubated with an affinity purified antibody against the collagen VI α3 chain followed by an Alexa 546 labeled secondary antibody (red). Nuclei were stained with DAPI (blue). d =  days after wounding. d =  dermis, e =  epidermis, g =  granulation tissue, st =  scar tissue. Bar, 200 µm.

**Figure 2:**
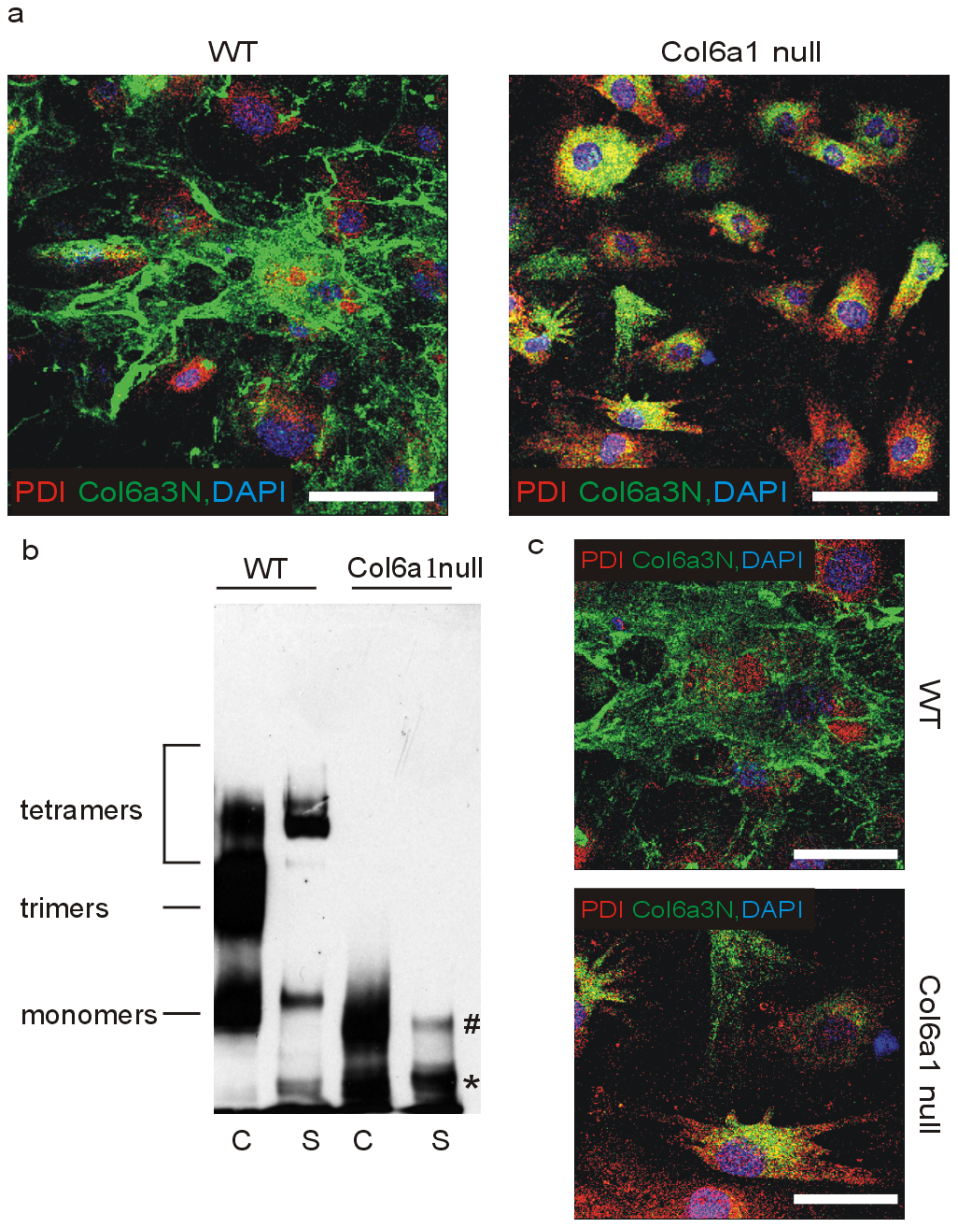
Analysis of the collagen VI α3 chain in primary fibroblast cultures from wild type and *Col6a1* null mouse skin. Cells were isolated from newborn wild type and *Col6a1* null mice and cultured for 4 days. (a) Immunostaining for the collagen VI α3 chain (green) and the endoplasmic reticulum marker PDI (red). Nuclei were stained with DAPI (blue). Bar, 100 µm. (b) Immunoblot analysis of collagen VI assembly in cell lysates (C) and supernatants (S). Cells were lysed with SDS-PAGE sample buffer, samples treated with 2 M urea and separated under non-reducing conditions on an agarose-polyacrylamide (0.5%/2.4%) composite gel. Immunoblots were developed with an antibody against the collagen VI α3 chain. (*) indicates the mobility of the single α3 chain, (#) indicates α3 chain dimers. (c) Higher magnification from (a). Bar, 50 µm.

To detect consequences of the lack of the collagen VI α1 chain on the expression of the classical α2 and α3 chains and the newly identified α4, α5, and α6 chains, we performed a comprehensive study of the distribution of the six collagen VI chains in wounds of wild type and *Col6a1* null mice using chain-specific affinity purified antibodies. During wound healing the α1, α2 and α3 chains were strongly expressed in wild type skin and could be detected already at day 4 (Fig. 1.; Fig. S1c). Throughout the healing process α1, α2 and α3 chains were mainly found just below the newly formed epidermis. The α2 and the α3 chains were detected by immunofluorescence labelling also in skin and wounds of *Col6a1* null mice, although at a lower level (Fig.1; Fig. S1c). The staining appeared patchy and irregular compared to that of wild type mice. This pattern was further investigated by immunofluorescence staining of primary mouse fibroblast cultures with an antibody against the collagen VI α3 chain, which revealed that in *Col6a1* null fibroblasts the α3 chain is still expressed but largely retained in the endoplasmic reticulum (Fig. 2a). By contrast, wild type fibroblasts secreted collagen VI and formed an extended extracellular network. Analysis of cell culture media from *Col6a1* null primary fibroblasts showed that some α3 chain was present as a single chain, indicating that a fraction the collagen VI α3 chains were secreted as individual molecules without forming heterotrimers (Fig. 2b).

The novel collagen VI α4, α5 and α6 chains displayed a more restricted expression. In unwounded wild type skin the α5 chain was localised around blood vessels [16] and its expression was increased during wound healing (Fig. S3). The α5 chain was mainly present below the granulation tissue. Interestingly, in the wound area the α5 chain was localised in the epineurium of newly formed nerves, but not around blood vessels (Fig. S4). The expression of the α6 chain was up-regulated during wound healing and was also detected below the granulation tissue (Fig. S5). Whereas labelling for the α5 chain started at day 7 of wound healing, the α6 chain was detected already at day 4. At later stages of wound healing, the staining for the α5 and α6 chains decreased. The α4 chain was not detected in mouse skin. *Col6a1* null skin was negative for all novel chains.

**Figure 3:**
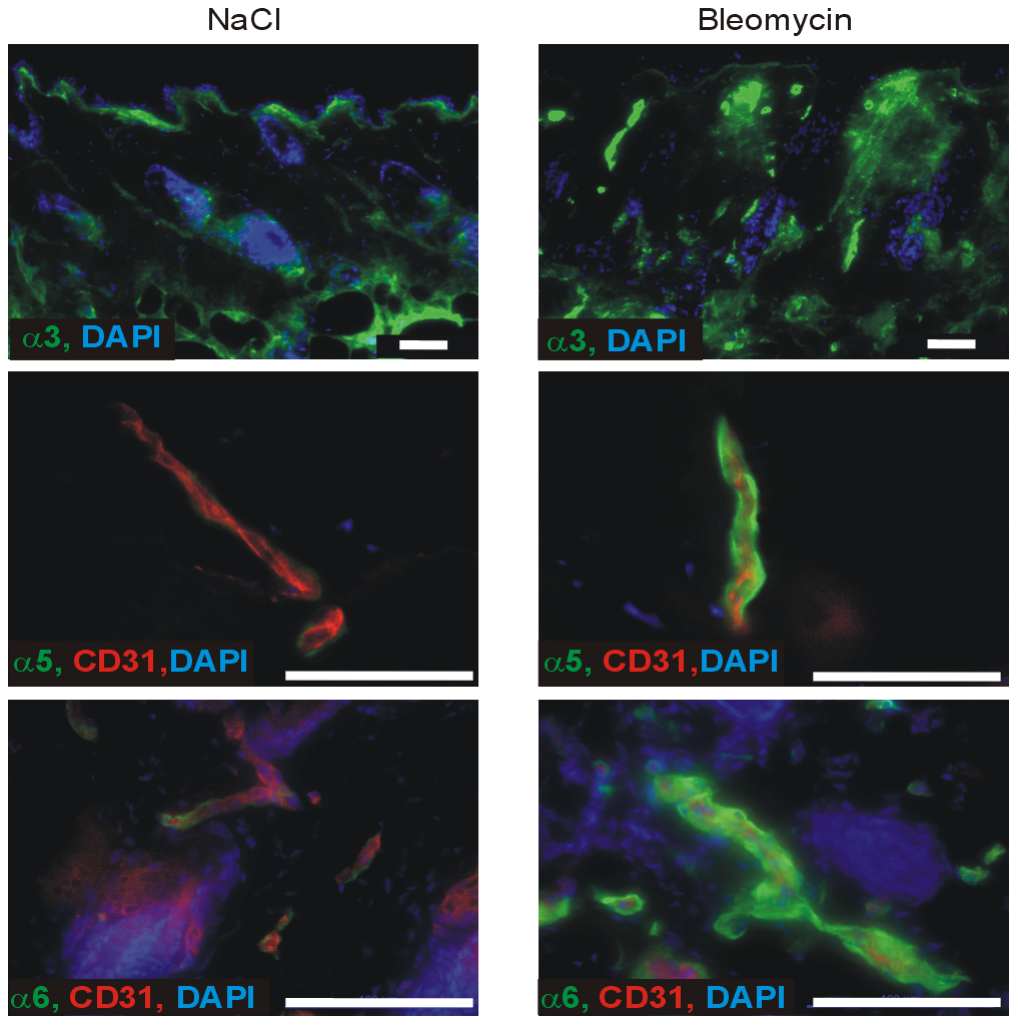
Collagen VI distribution in fibrotic skin lesions of wild type mice. Mice were treated for 4 weeks with bleomycin as described in Methods. NaCl injection served as control. Frozen sections were incubated with affinity purified antibodies against the collagen VI α3 (green), α5 (green) or α6 (green) chains. The sections stained for the α5 and α6 chains were co-stained with an antibody against the endothelial marker CD31 (red). Nuclei were stained with DAPI (blue). Bar, 50 µm.

As dysregulation of the tissue remodelling phase of wound healing results in fibrosis, we also studied the expression of the collagen VI chains in fibrotic skin induced by local bleomycin injection. Here, as in wound healing, the α3 chain was strongly expressed in the fibrotic dermis. However, in contrast to wounds where the α5 chain was absent from blood vessels, this chain was up-regulated in the blood vessels in the fibrotic dermis. As in wounds, the expression of the α6 chain was also up-regulated in in the blood vessels in the fibrotic dermis (Fig.3).

### The collagen VI α3 chain in skin is proteolytically processed

**Figure 4:**
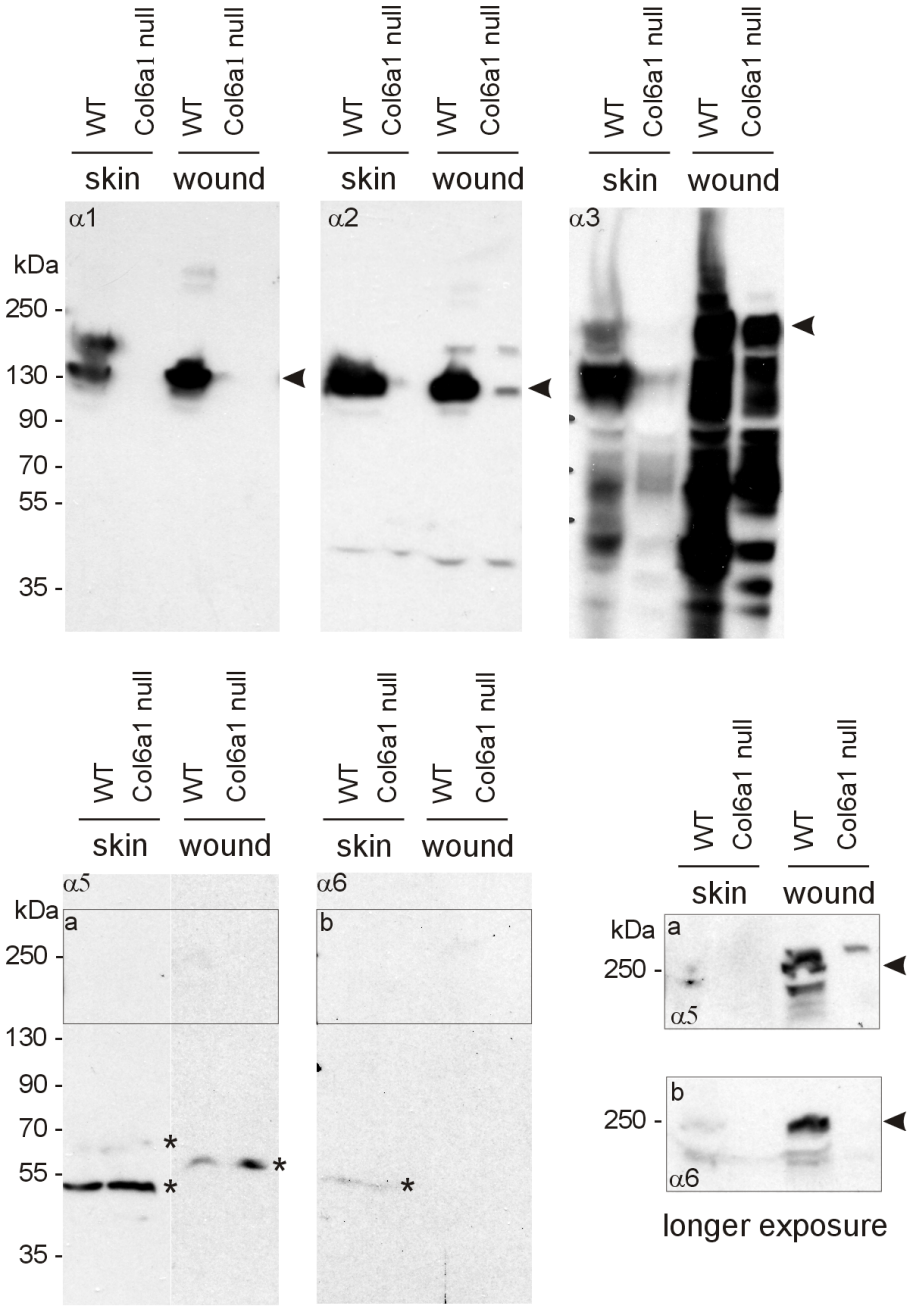
Collagen VI in extracts of unwounded skin and wounds derived from wild type and *Col6a1* null mice. Extracts from unwounded skin and wounds were subjected to SDS-PAGE on 4–12% polyacrylamide gradient gels under reducing conditions, proteins transferred to a membrane and detected with affinity purified antibodies against the collagen VI α1, α2, α3, α5 and α6 chains. Boxed areas a and b on the right show a longer exposure. Arrows indicate the position of the full length proteins, asterisks indicate artefact bands.

The α1, α2 and α3 chains were detected by immunoblot of extracts from both unwounded skin and the wound area (Fig. 4). In *Col6a1* null mice, not only the α1 chain but also the α2 chain was not detected in extracts from unwounded skin, however, a weak α2 chain signal was seen in extracts of wounds of these mice at day 7. The lacking signal for the α2 chain in unwounded skin is in contrast to the immunohistochemical analysis, probably because the amount of this chain extracted for immunoblots was below the detection limit. The α2 chains from wild type and *Col6a1* null mice migrated with the same expected mobility. In contrast, the α3 chain was detected in both uninjured skin and wounds at day 7, but was extensively degraded in unwounded skin of wild type mice and even more so in wounds of wild type and *Col6a1* null mice. Immunoblotting revealed a ladder of bands ranging from the full-length protein to 35 kDa fragments (Fig. 4). Interestingly, wound extracts contained more α3 chain and α3 chain fragments than extracts of unwounded skin, indicating an increased synthesis or greater solubility of collagen VI in wounds. The extracted material may represent tetramers that have not yet been assembled into fibrils or molecules that are being degraded due to high protein turnover. Also wound extracts from *Col6a1* null mice contained more α3 chain, than extracts of unwounded skin. This material probably represents a soluble intracellular pool of α3 chain in the *Col6a1* null fibroblasts (Fig. 4).

The full-length α5 and α6 chains gave only weak bands in immunoblots of extracts of unwounded skin of wild type mice. In wound extracts at day 7 the signals for the full-length chains were stronger, but absent in extracts from *Col6a1* null mice except for a weak band for the α5 chain (Fig. 4).

### Altered tensile strength and collagen fibril and basement membrane architecture in *Col6a1* null mice

**Figure 5:**
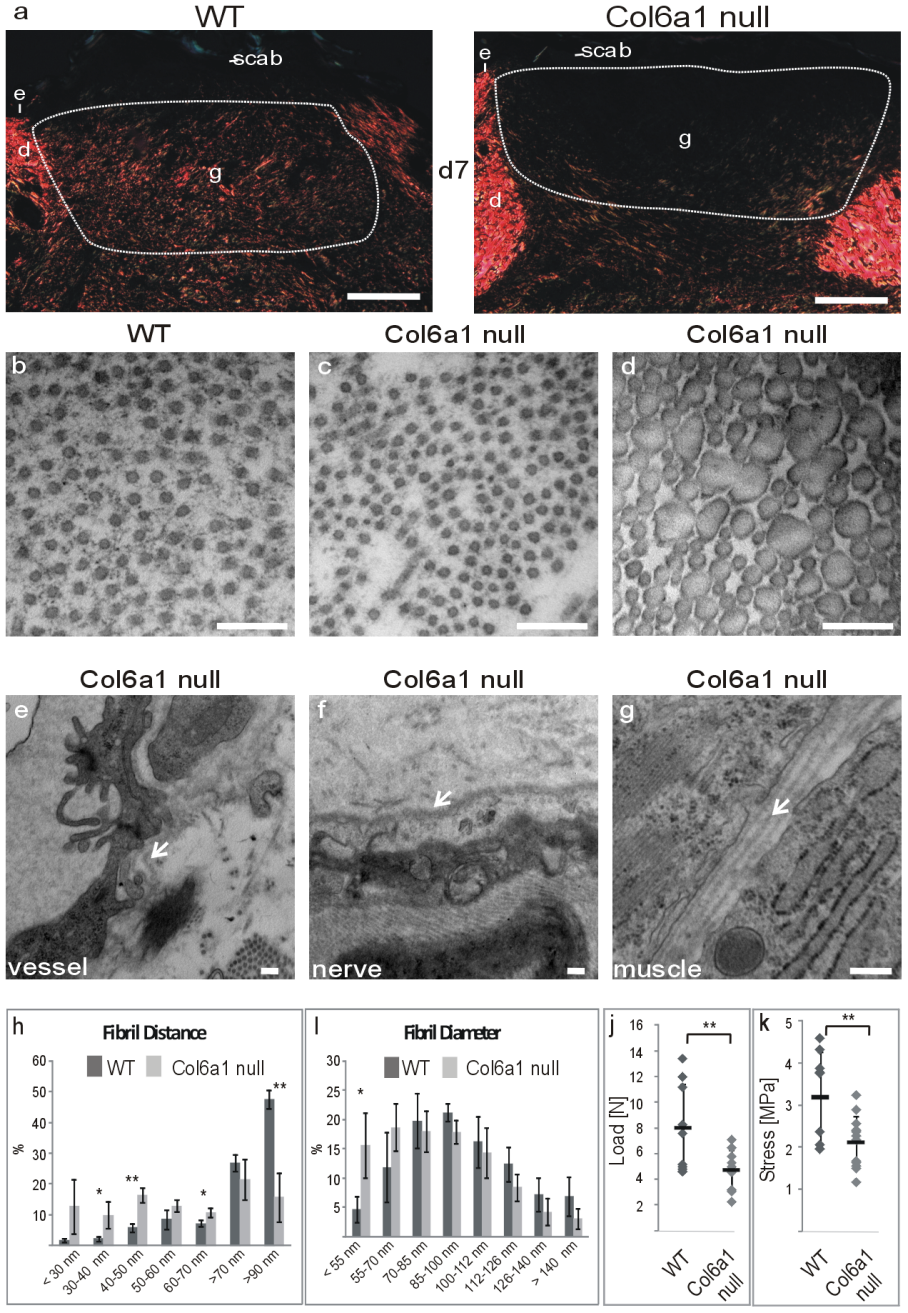
Analysis of collagen fibrils in wounds of wild type and *Col6a1* null mice and of basement membranes and tensile strength in skin of *Col6a1* null mice. (a) Picrosirius red staining of day 7 wounds. d =  dermis, e =  epidermis, g =  granulation tissue, st =  scar tissue. Bar, 200 µm. (b–g) Transmission electron microscopy of day 7 wounds (b–d) and of blood vessels (e), nerves (f) and muscle (g) in unwounded *Col6a1* null skin. Arrows indicate duplicated basement membranes. (h, i) Quantification of the distance between fibrils (wt = 903; *Col6a1* null  = 1776) (h) and of fibril diameter (wt = 890; *Col6a1* null  = 1091) (i) in areas from (b) and (c), (two electron micrographs from two animals per genotype were used for quantification). Load (j) and stress (k) are significantly reduced in unwounded *Col6a1* null skin. *<0.05, **<0.005, ***<0.0005. Bar, 250 nm.

Collagen VI microfibrils are connected to large collagen fibrils [21] and are thought to regulate their formation [22]. We therefore stained wounds of wild type and *Col6a1* null mice with antibodies against collagen I, but the overall distribution of this collagen was similar between genotypes (not shown). Picrosirius red staining and polarization microscopy can reveal changes in the properties of collagen fibrils. Indeed, a reduced birefringence was observed at day 7 in wounds of *Col6a1* null mice (Fig. 5a). This reduction was not apparent anymore at day 10 of wound healing (not shown).

Confirming this result, analysis of the collagen I fibril architecture by electron microscopy revealed changes in the extracellular matrix between the fibrils in wounds of the *Col6a1* null mice at day 7 (Fig. 5b–d). At central areas of the wound, the amount of fine microfibrillar structures interwoven between the collagen I fibrils was reduced and the collagen I fibrils were more densely packed (Fig. 5c, h). In contrast, the differences in fibril diameter distribution were marginal (Fig. 5i). In addition, in more peripheral areas of the wound where the collagen I fibrils had larger diameters, an irregular fusion of fibrils was often seen (Fig. 5d). No obvious changes in collagen fibril architecture were detected in unwounded skin from mice of the two genotypes (not shown). However, to investigate if the lack of collagen VI alters the tensile strength of unwounded skin, we performed mechanical tests on skin of wild type and collagen VI null mice. When the skin was stretched, the ultimate load and stress were significantly lower in collagen VI null mice in indicating that their skin is less strong. Moreover, in addition to abnormalities in collagen I fibril architecture in wounds, electron micrographs of unwounded skin revealed that the basement membranes of blood vessels and nerves as well as of adjacent muscles were sometimes duplicated in *Col6a1* null mice (Fig. 5e–g).

## Discussion

Collagen VI is thought to contribute to tissue remodelling [23], [24] and in addition to the obvious muscular pathologies, mutations in human collagen VI genes also often lead to keloid formation and other skin related phenotypes. *Col6a1* null mice serve as a well-established model for the muscle phenotypes, but have not been studied with regard to skin changes. In a first step we characterized the expression of collagen VI chains in mouse skin. We then performed wound healing experiments in skin of wild type and *Col6a1* null mice to assess whether this mouse model is also useful to assess the relevance of the classical collagen VI for skin development and for tissue reconstitution following injury. Since absence of the collagen VI α1 chain results in the failure to form the classical α1α2α3 trimer of collagen VI, we moreover sought to assess compensatory expression of the recently identified α4, α5 and α6 chains of collagen VI.

In human wounds, the classical α1 α2 and α3 chains were studied at the mRNA level revealing increased expression in fibroblast-like cells and in endothelial cells of newly formed vessels [25]. Collagen VI gene expression was not detected in smooth muscle cells or in myoepithelial cells of eccrine glands. We could show that in granulation tissue of wounds of wild type mice the classical α1, α2 and α3 chain-containing collagen VI was more strongly expressed than in unwounded skin. The widespread deposition of the protein in dermis indicates that collagen VI is abundantly secreted by fibroblasts. In addition, in wounded skin of wild type mice the α5 chain was up-regulated in the epineurium of newly formed nerves and the α6 chain in the tissue below the wound, but not within the granulation tissue. These results indicate that collagen VI is involved in the wound healing process and that the novel chains could play a more specific role than the broadly expressed classical ones. Up-regulation of collagen VI containing the classical chains was also detected in spontaneously fibrotic skin of tight skin (Tsk+/−) mice [26] and in bleomycin induced lung fibrosis [27]. Indeed, using the bleomycin model of skin fibrosis, we also found up-regulation of the α3 chain in the dermis and of the α5 and α6 chains in blood vessels, indicating that collagen VI is generally up-regulated in fibrotic tissue. Similarly, the α6 chain is expressed at higher levels in fibrotic muscle of Duchenne muscular dystrophy patients [28]. Interestingly, it was recently shown that a proteolytic fragment of collagen VI α1 chain is significantly elevated in the serum of patients with chronic obstructive pulmonary disease or idiopathic pulmonary fibrosis [29] and in a rat model of liver fibrosis [30]. However, in contrast to the hypertrophic scars or keloids occurring in patients with a collagen VI myopathies, we did not observe such disturbed wound healing in the *Col6a1* null mice. Most likely this can be explained by the fact that mice have a lesser tendency to overshooting wound healing than humans [31].

Interestingly, a patchy immunofluorescence staining for the α2 and α3 chains was observed in *Col6a1* null mice, and by immunoblot analysis we detected a more pronounced α3 chain degradation in wound extracts of wild type and *Col6a1* null mice. This is in contrast to the expectation that assembly and secretion of all collagen VI chains is abolished in *Col6a1* null mice, based on the fact that the α1 chain is absolutely required for the assembly of triple helical collagen VI molecules [32]. The distribution of the α3 chain is reminiscent of the reduced and patchy collagen VI α3 chain staining seen in a patient with UCMD carrying a mutation in *COL6A1* [28]. Furthermore, the intracellular accumulation of the α3 chain in *Col6a1* null fibroblasts correlates with the increased intracellular collagen VI labelling that was seen in cultures from UCMD patients [5] and the accumulation of the α1 chain in collagen VI α3 chain mutant mouse fibroblasts [33], indicating that synthesis of individual collagen VI chains is independent. Co-immunolabelling with an antibody against the endoplasmic reticulum (ER) protein PDI indicated that the α3 chain was retained in this subcellular compartment, as has been described for UCMD patients [34]. By immunoblot we could show that the formation of collagen VI tetramers is abolished in *Col6a1* null mice, thereby blocking collagen VI microfibril assembly. However, intracellular and secreted proteolytic fragments of the other collagen VI α chains are present and may contribute to pathogenic mechanisms by a deleterious action inside or outside of the cell. Indeed, recently a cleavage product of the collagen VI α3 chain, named endotrophin, was shown to augment fibrosis, angiogenesis, and inflammation through recruitment of macrophages and endothelial cells and was associated with aggressive mammary tumor growth and metastasis. These effects were partially mediated through enhanced TGF-β signaling, which contributes to tissue fibrosis [35]. In our model, more collagen VI and its fragments are extracted from granulation tissue than from unwounded skin, indicating either an increased synthesis or a decreased anchorage in the newly formed tissue. This may allow an increased diffusion into neighbouring tissues, thereby promoting TGF-β signaling.

Although collagen VI, predominantly in its classical form, is strongly expressed in wounds, the consequences of its absence are not overt. Immunofluorescence staining for wound healing markers such as α-smooth muscle actin, desmin, the F4/80 epitope or CD31 and for several extracellular matrix proteins and collagen VI binding partners did not show marked differences between wild type and *Col6a1* null mice (not shown). Only when collagen fibrils at day 7 of wound healing were stained with picrosirus red and visualized by polarization microscopy, a clear difference between wild type and *Col6a1* null mice was seen. The reason for this difference became obvious when the collagen I fibrils in day 7 wounds were visualized in greater detail by electron microscopy. A larger proportion of the fibrils were closely spaced in the *Col6a1* null mice than in wild type mice, indicating that collagen VI deficiency alters matrix architecture and possibly biomechanical properties. Similar ultrastructural alterations were also seen in tendons of mice deficient for either collagen VI α1 or α3 chains [33], [36]. In *Col6a1* null tendons the diameter distribution of collagen I fibrils was significantly shifted towards thinner fibrils. An analysis of fibril density (number/area unit) demonstrated a ∼2.5 fold increase in the *Col6a1* null versus wild type tendons and *Col6a1* null tendons displayed reduced biomechanical strength and stiffness [36], which corresponds to the reduced ultimate load and stress of *Col6a1* null skin in stretching experiments shown here (Fig. 5). Interestingly, ultrastructurally abnormal collagen I fibrils were observed in tendon, but not in cornea, of *Col6a1* null mice, indicating a tissue-specific action of collagen VI on collagen I fibrillogenesis [33]. Possibly the role of collagen VI is more pronounced in tissues which are exposed to mechanical stress. Nevertheless, a recent ultrastructural analysis of the skin of a patient with BM carrying a mutation in the collagen VI α2 chain revealed variations in size of collagen I fibrils, flower-like cross sections of collagen I fibrils, as well as thickening and duplication of vascular and nerve basement membranes in the skin [37] strikingly similar to the changes that we detected in *Col6a1* null mice. Indeed, this peculiar combination of signs was considered to be of diagnostic value. Interestingly, the unusual combination of basement membrane thickening and duplication was also detected in blood vessels of muscles of a myosclerosis patient carrying a mutation in the collagen VI α2 chain [3]. This indicates that although *Col6a1* null mice do not display an overt wound healing defect, some features seen in skin of collagen VI related myopathy patients are also present in *Col6a1* null mice. These mice are therefore the most relevant animal model available to study mechanistic aspects of the skin pathology in collagen VI related disease.

## Materials and Methods

### Ethics statement

This study was carried out in strict accordance with the German federal law on Animal Welfare, and the protocols were approved by the Landesamt für Natur, Umwelt und Verbraucherschutz Nordrhein-Westfalen (permit No. 8.87–51.04.20.09.338 for wound healing experiments; permit No. 8.87–50.10.31.08.197 for bleomycin induced fibrosis experiments).

### Histology and morphometric analysis

Frozen sections (7 µm) were fixed with 2% paraformaldehyde and stained with haematoxylin and eosin (H/E) to determine the granulation tissue area and the distance between the ends of the panniculus carnosus as described [38] using the ImageJ software. Significance of differences was analyzed using the two-tailed t-test. All data were presented as the mean ±SD, a p value of <0.05 was considered significant. Staining with picrosirius red was used to examine collagen distribution and characteristics.

### Immunofluorescence microscopy

Immunofluorescence microscopy was performed on frozen sections (7 µm) of healthy or fibrotic skin and of wounds from wild type and *Col6a1* null mice [32]. Sections were fixed with 2% paraformaldehyde and treated with bovine testicular hyaluronidase. Primary antibodies were applied over night at 4°C followed by incubation with secondary AlexaFluor 546-conjugated goat anti-rabbit IgG (Molecular Probes), AlexaFluor 488-conjugated goat anti-rabbit IgG (Molecular Probes), AlexaFluor 546-conjugated goat anti-guinea pig IgG, or AlexaFluor 546-conjugated rat anti-mouse pig IgG (Molecular Probes). The antibodies raised against recombinant N-terminal fragments of the collagen VI α3, α5 and α6 chains have been described [15] and those against the collagen VI α1 and α2 chains were raised by immunization with recombinant C-terminal fragments of these chains. The ER marker protein disulfide isomerase (PDI) was detected with a purified anti-rabbit PDI antibody (Stressgene), the endothelial marker CD31 with a purified rat anti-mouse CD31 antibody (MEC13.3, BD-Pharmingen) and nerves with a purified rat anti-mouse neurofilament antibody (Dako). Nuclei were stained with DAPI (Sigma Aldrich).

### Protein extraction from skin and wounds

Skin and wound tissues were frozen in liquid nitrogen and pulverized by pestle and mortar, lysed in 50 mM Tris, pH 7.4, containing 150 mM NaCl, 10 mM MgCl_2_, 0.5 mM dithiothreitol, 1 mM EDTA, 10% glycerol, 2% SDS and 1% Triton-X100 together with protease inhibitors (complete, Roche), incubated at 70°C for 10 min and homogenized by a Rotor stator homogenizer. The samples were clarified by centrifugation at 4°C.

### Gel electrophoresis and immunoblot

For SDS-PAGE samples were reduced with 5% β-mercapthoethanol and subjected to electrophoresis on 4–12% (w/v) gradient polyacrylamid gels. Samples for electrophoresis on 0.5% agarose-2.4% polyacrylamide composite gels were pretreated with 2 M urea [39]. In both cases proteins were electrophoretically transferred to Immobilon-P membranes (Millipore). The collagen VI α3, α5 and α6 chains were detected with affinity purified antibodies raised by immunization with N-terminal recombinant fragments [15], the α1 and α2 chains with antibodies against C-terminal recombinant fragments. Secondary antibodies were conjugated with horseradish peroxidise and bands were visualized by chemiluminescence (SuperSignal West Pico, Pierce). The experiments were performed with extracts from three different animals per genotype. The individual animals gave similar results.

### Electron microscopy

Tissue was fixed in 2% paraformaldehyde, 2% glutardialdehyde in 0.1 M sodium cacodylate buffer, pH 7.4, at 4°C for 24–48 h. Post-fixation was in 2% osmium tetroxide buffered at pH 7.3 with sodium cacodylate for 2 h at 4°C. Biopsies were washed, stained in 1% uranyl acetate, dehydrated through a series of graded ethanols and embedded in epon resin. Semithin sections (500 nm) were cut with a glass knife on an ultramicrotome (Reichert) and stained with methylene blue. Ultrathin sections (70 nm) for electron microscopic evaluation were processed on the same microtome with a diamond knife and placed on copper grids. The ultrathin sections were analyzed with a Zeiss 902A Transmission Electron Microscope (Zeiss) and the TEM Imaging Platform iTEM Software (Soft Imaging Systems).

### Wound healing

Wounding and harvesting of wound tissue was performed as previously described [19], [20]. Briefly, mice were anesthetized by intraperitoneal injection with ketamine/xylazine and full-thickness wounds comprising the epidermis, dermis, subcutaneous fat and the panniculus carnosus muscle were created by a biopsy punch on the shaved backs. Animals were housed under a 12/12 hours light/dark cycle with free access to food and fresh water ad libitum. Mice were euthanized by carbon dioxide. For histological analysis, wounds were excised at different times after injury (4–14 days), bisected in the caudocranial direction, and the tissue was either fixed overnight in 4% formaldehyde or embedded in optimal cutting temperature compound (Tissue-Tek, Sakura).

### Bleomycin induced fibrosis

Experimental skin fibrosis was induced by repeated intradermal injection of bleomycin sulphate (100 µl, 1 mg/ml in 0.9% NaCl; Medac) into the back skin of six anaesthetized (isoflurane) and shaved 6 week old female C57Bl6/N mice on 5 days/week for 4 weeks as described [40]. Five control mice received intradermal injections of NaCl (100 µl, 0.9%). Animals were housed under specific pathogen-free conditions in a 12/12 hours light/dark cycle with free access to food and fresh water ad libitum. Mice were euthanized by carbon dioxide.

### Mechanical testing

The tensile strength of skin of 8-week-old wild type (n = 9) and *Col6a1* null (n = 12) mice was analysed using a material testing machine (Z2.5/TN1S; Zwick). Two stripes of back skin were dissected in hourglass-shaped form (width: 5 mm in middle and 10 mm at the ends, length: 25 mm) using a punch. These samples were fixed between two riffled clamps. After preloading (0.05 N, 0.1 mm/s) the skin was stretched until failure with a crosshead speed of 15 mm/s. To calculate the stress, skin thickness was assessed in sections prepared for histological analysis. Significance of differences was analyzed using the two-tailed t-test.

## Supporting Information

Figure S1 **Histological analysis of unwounded skin and wounds (a, b) and immunofluorescence analysis of collagen VI α1 and α2 chains (c).** (a) H/E staining of skin from 10 week-old wild type and *Col6a1* null mice. (b) H/E staining of wounds from wild type and *Col6a1* null mice at 4, 7, 10 and 14 days after wounding. (c) Sections from day 10 wounds were incubated with affinity purified antibodies against the collagen VI α1 and α2 chains, followed by Alexa 546 (red) labeled secondary antibody. Nuclei were stained with DAPI (blue). d =  dermis, e =  epidermis, g =  granulation tissue, hf =  hair follicle, st =  scar tissue, pc =  panniculus carnosus, arrow heads  =  ends of the panniculus carnosus. Bar, 100 µm.
(TIF)

Figure S2 **Quantification of the granulation tissue area (a) and of the distance between the ends of the panniculus carnosus (b).** The granulation tissue area and the distance between the ends of the panniculus carnosus from wild type and *Col6a1* null mice at 4, 6, 7, 8, 9 10 and 14 days after wounding was determined using the ImageJ software. The standard deviation is indicated. The significance was determined using a two-tailed t-test. There were no significant differences. N (wild type/*Col6a1* null)  = 6/6 (d4),  = 7/8(d6),  = 14/11 (d7),  = 4/6 (d8),  = 4/6 (d9),  = 6/8 (d10) and 7/9 (d14).
(TIF)

Figure S3 **Immunofluorescence analysis of the collagen VI α5 chain during wound healing**. Frozen sections of wounds from wild type and *Col6a1* null mice at days 4, 7, 10 and 14 after wounding were incubated with an affinity purified antibody against the collagen VI α5 chain followed by Alexa 546 labeled secondary antibody (red). Nuclei were stained with DAPI (blue). d =  dermis, e =  epidermis, g =  granulation tissue, st =  scar tissue. Bar, 200 µm.
(TIF)

Figure S4 **Immunofluorescence analysis of the collagen VI α5 chain in blood vessels and nerves**. (a) Wounds from wild type mice at day 7 after wounding were incubated with affinity purified antibody for collagen VI α5 chain (green). (b) Co-staining of collagen VI α5 chain (green) and the endothelial marker CD31 (red) in the wound margin. (c) Co-staining of collagen VI α5 chain (green) with the nerve marker neurofilament (red) in a tissue area below the granulation tissue. Primary antibodies were detected by Alexa 546 and Alexa 488 labeled secondary antibodies. Nuclei were stained with DAPI (blue). Bar, 100 µm.
(TIF)

Figure S5 **Immunofluorescence analysis of the collagen VI α6 chain during wound healing**. Frozen sections of wounds from wild type and *Col6a1* null mice at 4, 7, 10 and 14 days after wounding were incubated with an affinity purified antibody against the collagen VI α6 chain followed by Alexa 546 labeled secondary antibody (red). Nuclei were stained with DAPI (blue). d =  dermis, e =  epidermis, g =  granulation tissue, st =  scar tissue. Bar, 200 µm.
(TIF)

## References

[1] Jöbsis GJ, Keizers H, Vreijling JP, de Visser M, Speer MC, et al (1996) Type VI collagen mutations in Bethlem myopathy, an autosomal dominant myopathy with contractures. Nat Genet 14: 113–115 10.1038/ng0996-113 8782832

[2] Vanegas OC, Zhang R-Z, Sabatelli P, Lattanzi G, Bencivenga P, et al (2002) Novel COL6A1 splicing mutation in a family affected by mild Bethlem myopathy. Muscle Nerve 25: 513–519 10.1002/mus.10100 11932968

[3] Merlini L, Martoni E, Grumati P, Sabatelli P, Squarzoni S, et al (2008) Autosomal recessive myosclerosis myopathy is a collagen VI disorder. Neurology 71: 1245–1253 10.1212/01.wnl.0000327611.01687.5e 18852439

[4] Pepe G, de Visser M, Bertini E, Bushby K, Vanegas OC, et al (2002) Bethlem myopathy (BETHLEM) 86th ENMC International Workshop, 10–11 November 2000, Naarden, The Netherlands. Neuromuscul Disord 12: 296–305 10.1016/S0960-8966(01)00275-9 11801404

[5] Jimenez-Mallebrera C, Maioli MA, Kim J, Brown SC, Feng L, et al (2006) A comparative analysis of collagen VI production in muscle, skin and fibroblasts from 14 Ullrich congenital muscular dystrophy patients with dominant and recessive COL6A mutations. Neuromuscul Disord NMD 16: 571–582 10.1016/j.nmd.2006.07.015 16935502

[6] Lampe AK, Zou Y, Sudano D, O'Brien KK, Hicks D, et al (2008) Exon skipping mutations in collagen VI are common and are predictive for severity and inheritance. Hum Mutat 29: 809–822 10.1002/humu.20704 18366090

[7] Nadeau A, Kinali M, Main M, Jimenez-Mallebrera C, Aloysius A, et al (2009) Natural history of Ullrich congenital muscular dystrophy. Neurology 73: 25–31 10.1212/WNL.0b013e3181aae851 19564581

[8] Briñas L, Richard P, Quijano-Roy S, Gartioux C, Ledeuil C, et al (2010) Early onset collagen VI myopathies: Genetic and clinical correlations. Ann Neurol 68: 511–520 10.1002/ana.22087 20976770

[9] Whittaker CA, Hynes RO (2002) Distribution and Evolution of von Willebrand/Integrin A Domains: Widely Dispersed Domains with Roles in Cell Adhesion and Elsewhere. Mol Biol Cell 13: 3369–3387 10.1091/mbc.E02-05-0259 12388743 PMC129952

[10] Chu ML, Conway D, Pan TC, Baldwin C, Mann K, et al (1988) Amino acid sequence of the triple-helical domain of human collagen type VI. J Biol Chem 263: 18601–18606. 3198591

[11] Knupp C, Squire JM (2001) A new twist in the collagen story—the type VI segmented supercoil. EMBO J 20: 372–376 10.1093/emboj/20.3.372 11157744 PMC133475

[12] Bruns RR (1984) Beaded filaments and long-spacing fibrils: relation to type VI collagen. J Ultrastruct Res 89: 136–145. 6100555 10.1016/s0022-5320(84)80010-6

[13] Von der Mark H, Aumailley M, Wick G, Fleischmajer R, Timpl R (1984) Immunochemistry, genuine size and tissue localization of collagen VI. Eur J Biochem FEBS 142: 493–502. 10.1111/j.1432-1033.1984.tb08313.x 6432530

[14] Fitzgerald J, Rich C, Zhou FH, Hansen U (2008) Three novel collagen VI chains, alpha4(VI), alpha5(VI), and alpha6(VI). J Biol Chem 283: 20170–20180 10.1074/jbc.M710139200 18400749

[15] Gara SK, Grumati P, Urciuolo A, Bonaldo P, Kobbe B, et al (2008) Three novel collagen VI chains with high homology to the alpha3 chain. J Biol Chem 283: 10658–10670 10.1074/jbc.M709540200 18276594

[16] Gara SK, Grumati P, Squarzoni S, Sabatelli P, Urciuolo A, et al (2011) Differential and restricted expression of novel collagen VI chains in mouse. Matrix Biol J Int Soc Matrix Biol 30: 248–257 10.1016/j.matbio.2011.03.006 21477648

[17] Angelin A, Tiepolo T, Sabatelli P, Grumati P, Bergamin N, et al (2007) Mitochondrial dysfunction in the pathogenesis of Ullrich congenital muscular dystrophy and prospective therapy with cyclosporins. Proc Natl Acad Sci U S A 104: 991–996 10.1073/pnas.0610270104 17215366 PMC1783427

[18] Grumati P, Coletto L, Sabatelli P, Cescon M, Angelin A, et al (2010) Autophagy is defective in collagen VI muscular dystrophies, and its reactivation rescues myofiber degeneration. Nat Med 16: 1313–1320 10.1038/nm.2247 21037586

[19] Lucas T, Waisman A, Ranjan R, Roes J, Krieg T, et al (2010) Differential roles of macrophages in diverse phases of skin repair. J Immunol Baltim Md 1950 184: 3964–3977 10.4049/jimmunol.0903356 20176743

[20] Willenborg S, Lucas T, van Loo G, Knipper JA, Krieg T, et al (2012) CCR2 recruits an inflammatory macrophage subpopulation critical for angiogenesis in tissue repair. Blood 120: 613–625 10.1182/blood-2012-01-403386 22577176

[21] Wiberg C, Klatt AR, Wagener R, Paulsson M, Bateman JF, et al (2003) Complexes of matrilin-1 and biglycan or decorin connect collagen VI microfibrils to both collagen II and aggrecan. J Biol Chem 278: 37698–37704 10.1074/jbc.M304638200 12840020

[22] Minamitani T, Ikuta T, Saito Y, Takebe G, Sato M, et al (2004) Modulation of collagen fibrillogenesis by tenascin-X and type VI collagen. Exp Cell Res 298: 305–315 10.1016/j.yexcr.2004.04.030 15242785

[23] Khan T, Muise ES, Iyengar P, Wang ZV, Chandalia M, et al (2009) Metabolic dysregulation and adipose tissue fibrosis: role of collagen VI. Mol Cell Biol 29: 1575–1591 10.1128/MCB.01300-08 19114551 PMC2648231

[24] Pasarica M, Gowronska-Kozak B, Burk D, Remedios I, Hymel D, et al (2009) Adipose tissue collagen VI in obesity. J Clin Endocrinol Metab 94: 5155–5162 10.1210/jc.2009-0947 19837927 PMC2819828

[25] Oono T, Specks U, Eckes B, Majewski S, Hunzelmann N, et al (1993) Expression of type VI collagen mRNA during wound healing. J Invest Dermatol 100: 329–334. 8440917 10.1111/1523-1747.ep12470022

[26] Pablos JL, Everett ET, Harley R, LeRoy EC, Norris JS (1995) Transforming growth factor-beta 1 and collagen gene expression during postnatal skin development and fibrosis in the tight-skin mouse. Lab Investig J Tech Methods Pathol 72: 670–678. 7783425

[27] Specks U, Nerlich A, Colby TV, Wiest I, Timpl R (1995) Increased expression of type VI collagen in lung fibrosis. Am J Respir Crit Care Med 151: 1956–1964 10.1164/ajrccm.151.6.7767545 7767545

[28] Sabatelli P, Gualandi F, Gara SK, Grumati P, Zamparelli A, et al (2012) Expression of collagen VI α5 and α6 chains in human muscle and in Duchenne muscular dystrophy-related muscle fibrosis. Matrix Biol J Int Soc Matrix Biol 31: 187–196 10.1016/j.matbio.2011.12.003 PMC3315014 22226732

[29] Leeming DJ, Sand JM, Nielsen MJ, Genovese F, Martinez FJ, et al (2012) Serological investigation of the collagen degradation profile of patients with chronic obstructive pulmonary disease or idiopathic pulmonary fibrosis. Biomark Insights 7: 119–126 10.4137/BMI.S9415 23012495 PMC3448496

[30] Leeming DJ, Byrjalsen I, Jiménez W, Christiansen C, Karsdal MA (2013) Protein fingerprinting of the extracellular matrix remodelling in a rat model of liver fibrosis–a serological evaluation. Liver Int Off J Int Assoc Study Liver 33: 439–447 10.1111/liv.12044 23279004

[31] Khorshid FA (2005) Comparative study of keloid formation in humans and laboratory animals. Med Sci Monit Int Med J Exp Clin Res 11: BR212–219. 15990682

[32] Bonaldo P, Braghetta P, Zanetti M, Piccolo S, Volpin D, et al (1998) Collagen VI deficiency induces early onset myopathy in the mouse: an animal model for Bethlem myopathy. Hum Mol Genet 7: 2135–2140. 9817932 10.1093/hmg/7.13.2135

[33] Pan T-C, Zhang R-Z, Markova D, Arita M, Zhang Y, et al (2013) COL6A3 protein deficiency in mice leads to muscle and tendon defects similar to human collagen VI congenital muscular dystrophy. J Biol Chem 288: 14320–14331 10.1074/jbc.M112.433078 23564457 PMC3656288

[34] Allamand V, Merlini L, Bushby K, Consortium for Collagen VI-Related Myopathies (2010) 166th ENMC International Workshop on Collagen type VI-related Myopathies, 22-24 May 2009, Naarden, The Netherlands. Neuromuscul Disord NMD 20: 346–354 10.1016/j.nmd.2010.02.012 20211562

[35] Park J, Scherer PE (2012) Endotrophin - a Novel Factor Linking Obesity with Aggressive Tumor Growth. Oncotarget. 10.18632/oncotarget.796 PMC3681481 23455368

[36] Izu Y, Ansorge HL, Zhang G, Soslowsky LJ, Bonaldo P, et al (2011) Dysfunctional tendon collagen fibrillogenesis in collagen VI null mice. Matrix Biol J Int Soc Matrix Biol 30: 53–61 10.1016/j.matbio.2010.10.001 PMC3778658 20951202

[37] Hermanns-Lê T, Piérard GE, Piérard-Franchimont C, Delvenne P (2013) Dermal Ultrastructure in Collagen VI Myopathy. Ultrastruct Pathol. doi: 10.3109/01913123.2013.829151. 10.3109/01913123.2013.829151 24134684

[38] Aghdam SY, Eming SA, Willenborg S, Neuhaus B, Niessen CM, et al (2012) Vascular endothelial insulin/IGF-1 signaling controls skin wound vascularization. Biochem Biophys Res Commun 421: 197–202 10.1016/j.bbrc.2012.03.134 22503682

[39] Peacock AC, Dingman CW (1968) Molecular weight estimation and separation of ribonucleic acid by electrophoresis in agarose-acrylamide composite gels. Biochemistry (Mosc) 7: 668–674. 10.1021/bi00842a023 4868544

[40] Yamamoto T, Takagawa S, Katayama I, Yamazaki K, Hamazaki Y, et al (1999) Animal model of sclerotic skin. I: Local injections of bleomycin induce sclerotic skin mimicking scleroderma. J Invest Dermatol 112: 456–462 10.1046/j.1523-1747.1999.00528.x 10201529

