# Supplementary figures and images for: Col6a1 Null Mice as a Model to Study Skin Phenotypes in Patients with Collagen VI Related Myopathies: Expression of Classical and Novel Collagen VI Variants during Wound Healing
Source: PLoS One. 2014 Aug 26;9(8):e105686. doi: 10.1371/journal.pone.0105686 (PMC4144880; doi:10.1371/journal.pone.0105686)

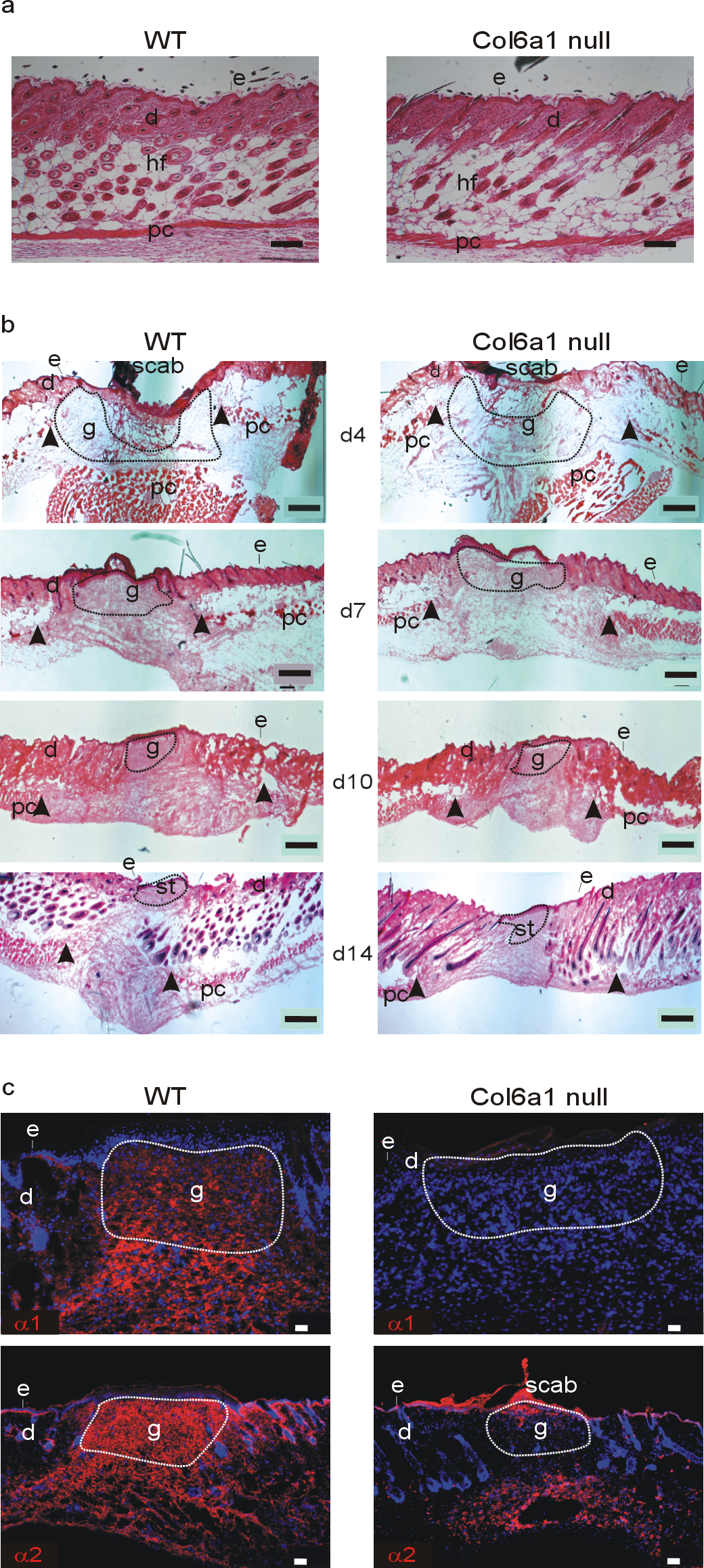

Supplement: Figure S1 — Histological analysis of unwounded skin and wounds (a, b) and immunofluorescence analysis of collagen VI α1 and α2 chains (c). (a) H/E staining of skin from 10 week-old wild type and Col6a1 null mice. (b) H/E staining of wounds from wild type and Col6a1 null mice at 4, 7, 10 and 14 days after wounding. (c) Sections from day 10 wounds were incubated with affinity purified antibodies against the collagen VI α1 and α2 chains, followed by Alexa 546 (red) labeled secondary antibody. Nuclei were stained with DAPI (blue). d = dermis, e = epidermis, g = granulation tissue, hf = hair follicle, st = scar tissue, pc = panniculus carnosus, arrow heads = ends of the panniculus carnosus. Bar, 100 µm. (TIF) [file pone.0105686.s001.tif]

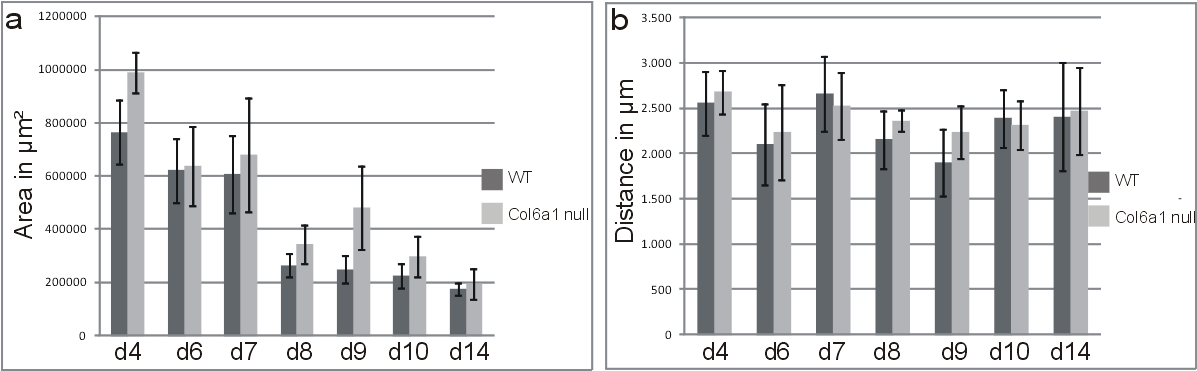

Supplement: Figure S2 — Quantification of the granulation tissue area (a) and of the distance between the ends of the panniculus carnosus (b). The granulation tissue area and the distance between the ends of the panniculus carnosus from wild type and Col6a1 null mice at 4, 6, 7, 8, 9 10 and 14 days after wounding was determined using the ImageJ software. The standard deviation is indicated. The significance was determined using a two-tailed t-test. There were no significant differences. N (wild type/Col6a1 null) = 6/6 (d4), = 7/8(d6), = 14/11 (d7), = 4/6 (d8), = 4/6 (d9), = 6/8 (d10) and 7/9 (d14). (TIF) [file pone.0105686.s002.tif]

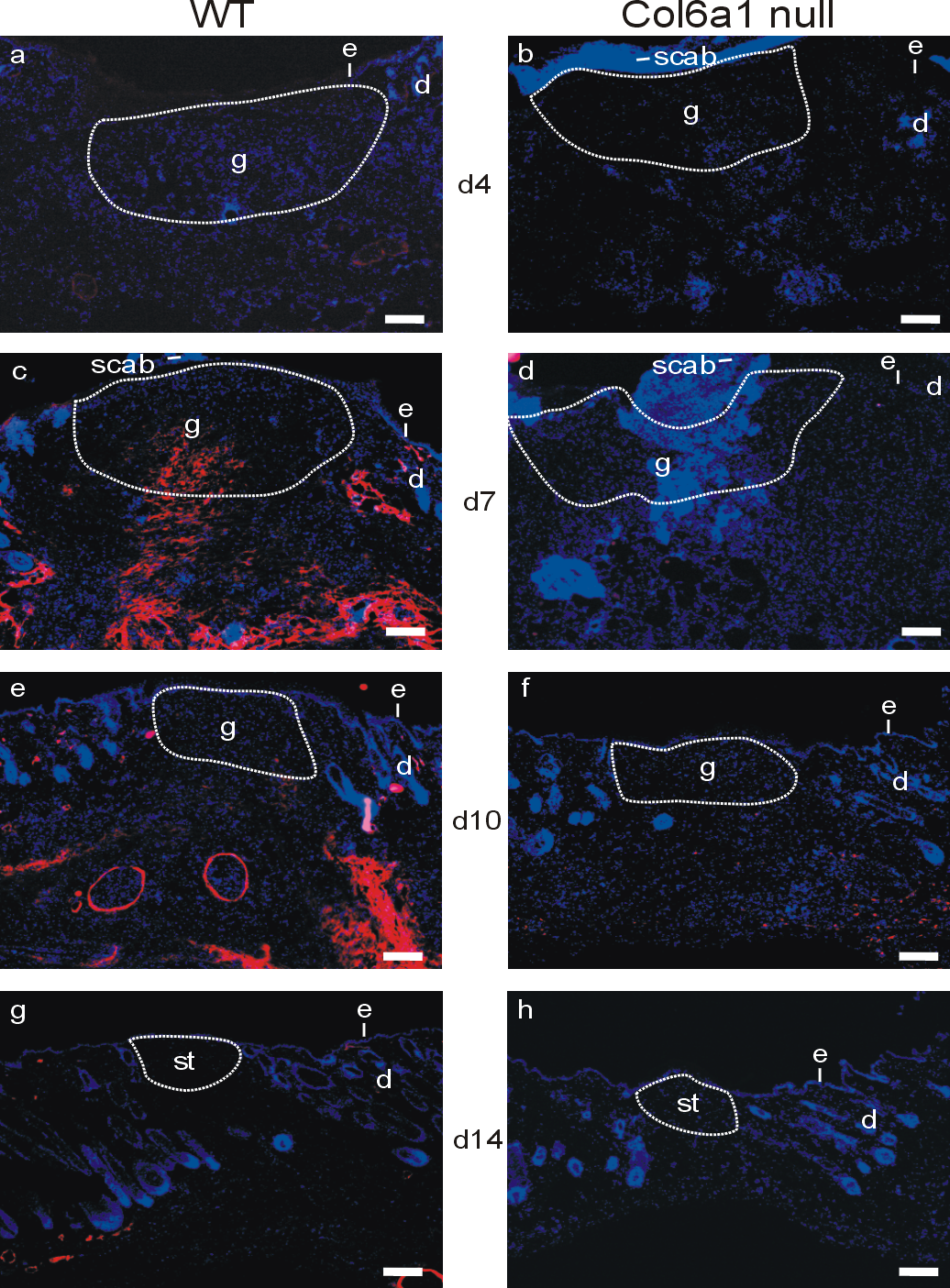

Supplement: Figure S3 — Immunofluorescence analysis of the collagen VI α5 chain during wound healing. Frozen sections of wounds from wild type and Col6a1 null mice at days 4, 7, 10 and 14 after wounding were incubated with an affinity purified antibody against the collagen VI α5 chain followed by Alexa 546 labeled secondary antibody (red). Nuclei were stained with DAPI (blue). d = dermis, e = epidermis, g = granulation tissue, st = scar tissue. Bar, 200 µm. (TIF) [file pone.0105686.s003.tif]

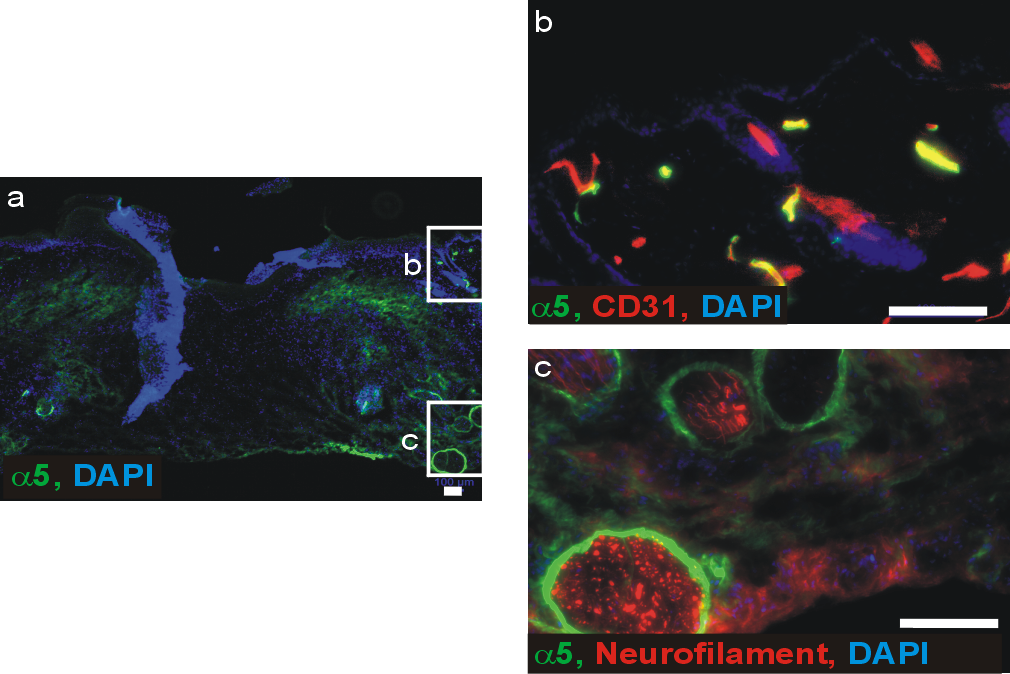

Supplement: Figure S4 — Immunofluorescence analysis of the collagen VI α5 chain in blood vessels and nerves. (a) Wounds from wild type mice at day 7 after wounding were incubated with affinity purified antibody for collagen VI α5 chain (green). (b) Co-staining of collagen VI α5 chain (green) and the endothelial marker CD31 (red) in the wound margin. (c) Co-staining of collagen VI α5 chain (green) with the nerve marker neurofilament (red) in a tissue area below the granulation tissue. Primary antibodies were detected by Alexa 546 and Alexa 488 labeled secondary antibodies. Nuclei were stained with DAPI (blue). Bar, 100 µm. (TIF) [file pone.0105686.s004.tif]

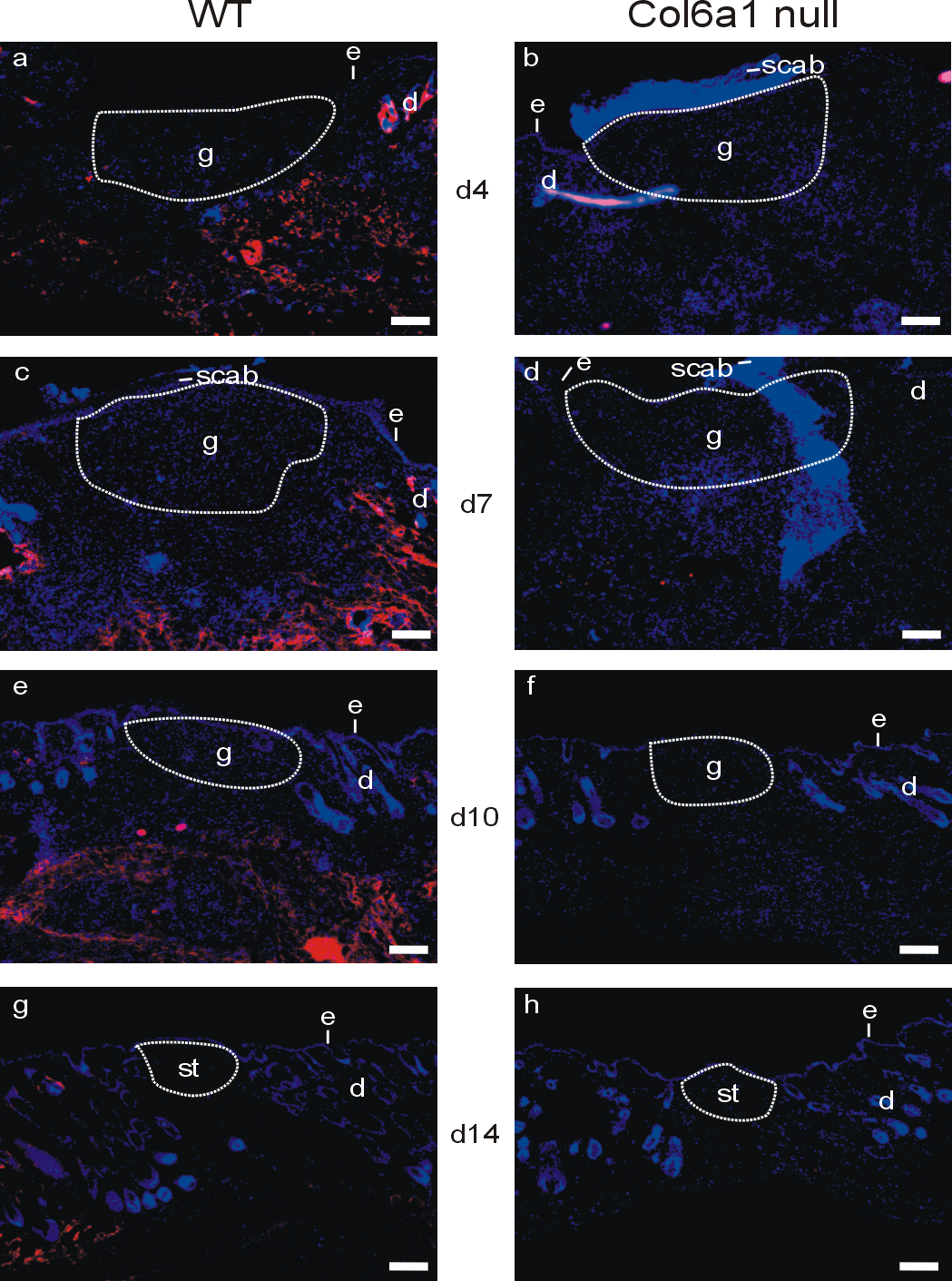

Supplement: Figure S5 — Immunofluorescence analysis of the collagen VI α6 chain during wound healing. Frozen sections of wounds from wild type and Col6a1 null mice at 4, 7, 10 and 14 days after wounding were incubated with an affinity purified antibody against the collagen VI α6 chain followed by Alexa 546 labeled secondary antibody (red). Nuclei were stained with DAPI (blue). d = dermis, e = epidermis, g = granulation tissue, st = scar tissue. Bar, 200 µm. (TIF) [file pone.0105686.s005.tif]
